# In this month’s Bulletin

**DOI:** 10.2471/BLT.25.000725

**Published:** 2025-07-01

In the editorial section, Nicolas Chartres et al. (414) argue that health implications of plastic pollution should figure more prominently in the global plastics treaty negotiations. Aklilu Azazh et al. (415) detail capacity requirements for local health policy development and implementation.

## Afghanistan, Democratic Republic of the Congo, Indonesia, Israel, Kenya, Kosovo, Nepal, Pakistan, Sri Lanka, Uganda, United States of America, West Bank and Gaza Strip

### Children and adolescents in humanitarian crises

Fiona E Douglas et al. (445–461) review the evidence for effective treatment of post-traumatic stress disorder.

## Australia

### Effective responses to avian influenza 

Karen Blaney et al. (437–444) describe protocols for poultry farms.

## Nigeria

### Deaths from severe acute malnutrition 

Emmanuel Grellety et al. (418–428) examine diagnostic criteria for hospitalized children.

## Global

### Predicting viral spread

Toshiaki R Asakura et al. (429–436) model international transmission of mpox.

### Adult mortality estimates

Ashira Menashe-Oren (462–462) et al. explain why improved measures are needed.

